# Functional Studies of ssDNA Binding Ability of MarR Family Protein TcaR from *Staphylococcus epidermidis*

**DOI:** 10.1371/journal.pone.0045665

**Published:** 2012-09-21

**Authors:** Yu-Ming Chang, Cammy K. -M. Chen, Yuan-Chih Chang, Wen-Yih Jeng, Ming-Hon Hou, Andrew H. -J. Wang

**Affiliations:** 1 Institute of Biological Chemistry, Academia Sinica, Taipei, Taiwan; 2 Core Facilities for Protein Structural Analysis, Academia Sinica, Taipei, Taiwan; 3 Institute of Cellular and Organismic Biology, Academia Sinica, Taipei, Taiwan; 4 University Center for Bioscience and Biotechnology, National Cheng Kung University, Tainan, Taiwan; 5 Biotechnology Center, National Chung Hsing University, Taichung, Taiwan; 6 Institute of Genomics and Bioinformatics, National Chung Hsing University, Taichung, Taiwan; 7 Department of Life Science, National Chung Hsing University, Taichung, Taiwan; Institute of Molecular and Cell Biology, Singapore

## Abstract

The negative transcription regulator of the *ica* locus, TcaR, regulates proteins involved in the biosynthesis of poly-*N*-acetylglucosamine (PNAG). Absence of TcaR increases PNAG production and promotes biofilm formation in *Staphylococci*. Previously, the 3D structure of TcaR in its apo form and its complex structure with several antibiotics have been analyzed. However, the detailed mechanism of multiple antibiotic resistance regulator (MarR) family proteins such as TcaR is unclear and only restricted on the binding ability of double-strand DNA (dsDNA). Here we show by electrophoretic mobility shift assay (EMSA), electron microscopy (EM), circular dichroism (CD), and Biacore analysis that TcaR can interact strongly with single-stranded DNA (ssDNA), thereby identifying a new role in MarR family proteins. Moreover, we show that TcaR preferentially binds 33-mer ssDNA over double-stranded DNA and inhibits viral ssDNA replication. In contrast, such ssDNA binding properties were not observed for other MarR family protein and TetR family protein, suggesting that the results from our studies are not an artifact due to simple charge interactions between TcaR and ssDNA. Overall, these results suggest a novel role for TcaR in regulation of DNA replication. We anticipate that the results of this work will extend our understanding of MarR family protein and broaden the development of new therapeutic strategies for *Staphylococci*.

## Introduction

*Staphylococci* are among the most common causes of bacterial infection in the community and pose a major danger to human health. *S. aureus* is the most well-known of the species which produce hospital- and community-acquired infections, with methicillin-resistant *S. aureus* presenting a serious public health threat [1]. *S. epidermidis* is the sister species of *S. aureus* which often causes infection in immunocompromised individuals or those following damage to the epithelium. Both of them produce biofilm to protect themselves from host immune system and enhance their resistance to antibiotic chemotherapy [2]. The key component of the biofilm extracellular matrix in *Staphylococci* is polysaccharide intercellular adhesin (PIA) [3], an essential factor in biofilm formation composed of homopolymer of β-1,6-linked N-acetylglucosamine (GlcNAc). The production of PIA depends on the expression of the *icaADBC* operon, and TcaR and IcaR are a weak and a strong negative regulator of transcription of the *ica* locus, respectively [4].

The transcription regulator TcaR is a member of the MarR family, and is involved in teicoplanin and methicillin resistance in *Staphylococci* [5]. The MarR family proteins function as regulators of protein expression and these regulated proteins confer resistance to multiple antibiotics, household disinfectants, organic solvent virulence factors, and oxidative stress agents [6], [7], [8], [9], [10], [11]. The crystal structures of a number of MarR family proteins have been reported, including MarR from *Escherichia coli* [12], OhrR from *Bacillis subtilis* [13], MexR from *Pseudomonas aeruginosa* [14], MarR from *Xanthomonas campestris* [15], SlyA from *Salmonella typhimurium* [16], AdcR from *Streptococcus pneumonia* [10] and TcaR, which is studied in this work, from *S. epidermidis* [17]. These structures revealed that MarR family proteins are all homodimers. The overall structure of each monomer could be divided into two functional domains, one is the dimerization domain and the other is the winged helix-turn-helix (wHTH) DNA binding domain. The N and the C-terminal α-helices (α1, 5, 6) of one monomer interdigitate with those of the other monomer to produce dimerization interaction. In addition, the wHTH DNA binding domain is composed of α2-α3-α4-βA-W1-βB which adopts the winged-helix-fold, and the amino acid sequences of this domain are highly conserved.

As the MarR-type proteins can act as positive, negative, or bifunctional regulators, TcaR also acts as a multi-functional regulator. It is not only as a regulatory factor to affect the transcription of *icaADBC* [4], the first regulator reported for cell wall-anchored proteins (SpA and sasF), but also as the regulator of *sarS* [18], [19]. We previously described the 3D structures of TcaR in its apo form and in complex with salicylate as well as several aminoglycoside and β-lactam antibiotics [17]. In this research, comparison of the native TcaR structures and those in complexes indicated that the regulation mechanism involves a large conformational shift in the DNA binding lobe. Several antimicrobial compounds inhibited TcaR–DNA interaction and further induced biofilm formation in *S. epidermidis.* In the present study, we found that TcaR could interact with ssDNA and the result demonstrated that TcaR shows a stronger preference toward GC-rich ssDNA than to dsDNA by using EMSA, CD, and Biacore experiments. However, the detailed mechanism of the interaction between TcaR and ssDNA still remains to be elucidated. In order to investigate the regulation mechanism of the ssDNA binding ability of TcaR, we applied electron microscopy (EM) technique to reveal TcaR-ssDNA complex. Furthermore, we clarified the role of TcaR-ssDNA interaction by *in vitro* replication assay and *in vivo* plaque assay. Taken together, these results provide an in-depth investigation on the multiple functions of TcaR in *S. epidermidis*.

## Results and Discussion

### Strong TcaR Binding to ssDNA Oligomers Revealed by EMSA

**Figure 1:**
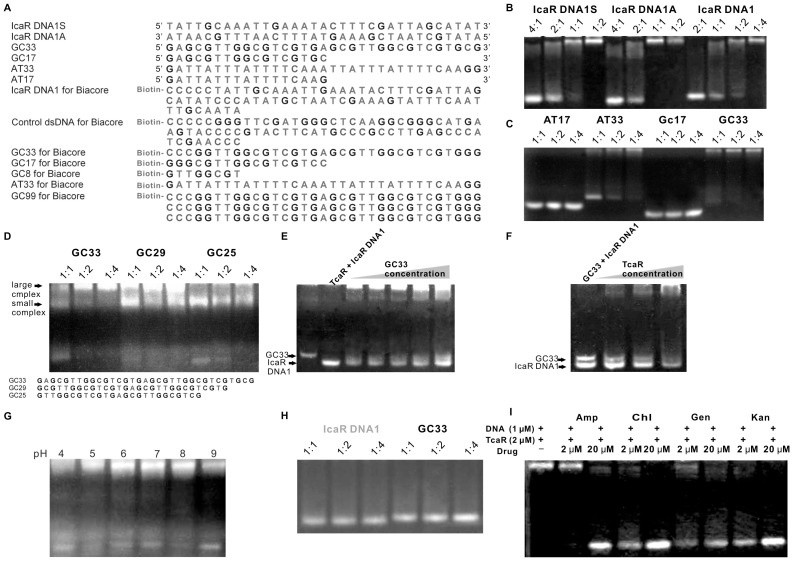
EMSA analysis of TcaR. (A) Sequences of the oligonucleotide probes used in EMSA and Biacore. IcaR DNA1 is an *ica* promoter fragment. Control dsDNA is the GC rich fragment of *IcaA* gene. IcaR DNA1S and IcaR DNA1A are sense and antisense strand of IcaR DNA1, respectively. AT17, AT33, GC8, GC17, GC33 and GC99 are 17-mer AT-rich ssDNA, 33-mer AT-rich ssDNA, 8-mer GC-rich ssDNA, 17-mer GC-rich ssDNA, 33-mer GC-rich ssDNA and 99-mer GC-rich ssDNA fragments, respectively. (B) EMSA analysis of the binding of TcaR protein to ssDNA (IcaR DNA 1S, IcaR DNA 1A) and dsDNA (IcaR DNA1) fragments with different DNA ratio. DNA probes were mixed with TcaR (dimer) with the molar ratio of 4∶1, 2∶1, 1∶1, 1∶2, and 1∶4. (C) EMSA analysis of the binding of TcaR protein to two 17-mer (AT17, GC17) and two 33-mer (AT33, GC33) ssDNA fragments with different DNA ratio. Molar ratios of DNA:TcaR were 1∶1, 1∶2, and 1∶4. (D) EMSA of TcaR binding to GC-rich DNA probes with different lengths. (E) EMSA analysis of the binding of TcaR protein to dsDNA (IcaR DNA1) in the present of competitor from ssDNA(GC33). IcaR DNA1 probe duplex of 1 µM was pre-incubated with 2 µM TcaR (dimer) at room temperature for 15 min before mixing with increasing concentration of GC33 ssDNA, followed by the same procedure as described in the legend to Figure 1B. (F) Competition experiment was carried out to compare the binding strength of TcaR to ssDNA and to dsDNA. In the EMSA analysis, 1 µM IcaR DNA1 probe duplex was pre-incubated with 1 µM GC33 ssDNA fragment for 15 min at room temperature before mixing with TcaR protein of increasing concentration. (G) The effects of pH to the TcaR binding to ssDNA GC33 in EMSA experiment. Molar ratio of GC33:TcaR was 1∶2. (H) EMSA analysis of mutant TcaR protein binding to ssDNA (GC33) and dsDNA (IcaR DNA1) fragments with different protein–DNA ratio. (I) EMSA analysis of the binding of TcaR protein to ssDNA(GC33) in the present of different types of antibiotics. GC33 ssDNA probe of 1 µM was preincubated with 2 µM TcaR (dimer) at room temperature for 15 min before mixing with 2 µM or 20 µM antibiotics, followed by the same procedure as described in the legend to Figure 1B.

TcaR is known to bind and regulate the *ica* promoter [4]. We previously identified that TcaR most strongly interacts with IcaR DNA1 (a 33-mer pseudo-palindromic sequence containing consensus sequence **TTNNAA**) compared with other designed IcaR DNA fragments [17]. However, when using the sense strand of IcaR DNA1 (IcaR DNA1S) and the antisense strand of IcaR DNA1 (IcaR DNA1A) (Figure 1A) as controls in electrophoretic mobility shift assays (EMSA), the result demonstrated that TcaR shows a stronger preferences toward ssDNA fragments (IcaR DNA1S and DNA1A) (Figure 1B). To determine the type and length of the TcaR-binding site on ssDNA, a series of GC-rich and AT-rich ssDNA segments were designed (Figure 1A) [20], [21]. Their TcaR binding ability was tested using EMSA. As shown in Figure 1C, TcaR does not significantly interact with 17-mer GC-rich (GC17) and AT-rich (AT17) ssDNA oligomers, but shows strong interaction with 33-mer GC-rich (GC33) and AT-rich (AT33) ssDNA sequences with a preference toward the 33-mer ssDNA sequence with a molar ratio of 1∶1. Thus, we suggest that TcaR prefers binding to the 33-mer ssDNA.

In order to evaluate the minimal DNA binding length of TcaR, GC-rich fragments of different lengths were designed. As seen in Figure 1D, GC-rich fragments with 33, 29, and 25 bases showed similar binding strength to TcaR; with TcaR forming a large, apparently multimeric complex with GC33, a small complex with GC25, and both small and large complexes with GC29 in EMSA. These results indicated that the minimal observed ssDNA fragment size to allow TcaR binding ranges between 17 to 25-mer; providing useful information for the design of a DNA fragment with precise length suitable for crystal packing. Up to now, only three MarR family protein complex structures have been reported, and the first one is complexed with dsDNA [13], [16], [22]. The second one is complexed with salicylate [12], [17], [22], [23] and we discovered the third case which is complexed with antibiotics [17]. We have already obtained TcaR-ssDNA crystals and collected X-ray diffraction data to 3.6 Å resolution at SPring-8 (Hyogo, Japan), beamline BL12B2. However, the phase problem is still the main challenge and the works are currently under progress.

Moreover, to investigate whether TcaR preferentially binds to ssDNA or dsDNA, the ability of ssDNA to compete with the TcaR-dsDNA complex was evaluated. For the competition assay, the IcaR DNA1 probe was preincubated with TcaR (dimer) protein to allow formation of the dsDNA-TcaR complex prior to mixing with increasing amounts of single-stranded GC33 DNA. It has been known that ssDNA products have lower migration velocity compared to its dsDNA counterparts in polyacrylamide electrophoresis [24], [25]. As shown in Figure 1E, ssDNA, as a competitor, interfered the binding of TcaR to the dsDNA, suggesting a binding preference for ssDNA. To further confirm this result, IcaR DNA1 and GC33 ssDNA oligomers were mixed, and their interaction strengths with TcaR were compared using EMSA (Figure 1F). Findings indicated that increasing the concentration of TcaR produces a ssDNA band shift greater than that for dsDNA, confirming a stronger interaction between TcaR and ssDNA. Moreover, to investigate possible pH effect of ssDNA binding activity of TcaR, a series of buffers with increasing pH were tested for their potential interfere in TcaR-ssDNA binding. As shown in Figure 1G, the EMSA results showed that TcaR had a strongest affinity for GC33 at pH 8.0 and the affinity was reduced by decreasing pH. Consequently, the result indicates that the ssDNA binding activity of TcaR is pH-dependent.

To clarify whether the ssDNA binding site of TcaR is close, or identical, to the dsDNA binding site, a TcaR quadruple mutant (4 positively charged amino acids responsible for DNA binding mutated to alanines to produce R71A/K73A/R93A/K95A) [17] was designed and its ssDNA and dsDNA binding ability tested. As seen in Figure 1H, the mutant failed to interact with either dsDNA or ssDNA. This indicated that these amino acids are essential for binding in both ssDNA and dsDNA.

### Binding of Antibiotics Abolished ssDNA Binding of TcaR

The MarR protein of *E. coli* is a multidrug binding transcription regulator. A wide range of compounds, including 2,4-dinitrophenol, plumbagin, menadione, and salicylate, attenuate and inhibit its association with cognate DNA [26]. In our previous study, salicylate and multiple antibiotics interfered with the transcriptional repressor activity of TcaR [17]. These findings prompted the current investigation into the possible effects of antibiotics on the ssDNA binding ability of TcaR. Here, to investigate the possible effect of some drugs on TcaR, four compounds were tested for their potential inhibition on TcaR-ssDNA interaction. These include one beta-lactam antibiotics (ampicillin) that contain a β-lactam nucleus in their molecular structure and act by inhibiting the synthesis of the peptidoglycan layer of bacterial cell walls, two aminoglycoside antibiotics (kanamycin and gentamicin) that composed of several sugar groups and amino groups, and bacteriostatic antimicrobial (chloramphenicol) which is considered as a prototypical broad-spectrum antibiotic.

As shown in Figure 1I, kanamycin, chloramphenicol and gentamycin interfered with the ssDNA (GC33) binding activity of TcaR at a concentration of 2 µM and this effect was more pronounced at a higher concentration, suggesting that antibiotics inhibit formation of the TcaR-ssDNA complex. Results indicated that ampicillin also antagonized the ssDNA binding activity of TcaR at a concentration of 20 µM. This result is consistent with the observation seen in SPR, as discussed below. Taken together, we believe that diverse kinds of antibiotics may interact with TcaR to regulate its ssDNA binding ability.

### SPR and CD Spectral Analysis of TcaR Bound to ssDNA and dsDNA

**Figure 2:**
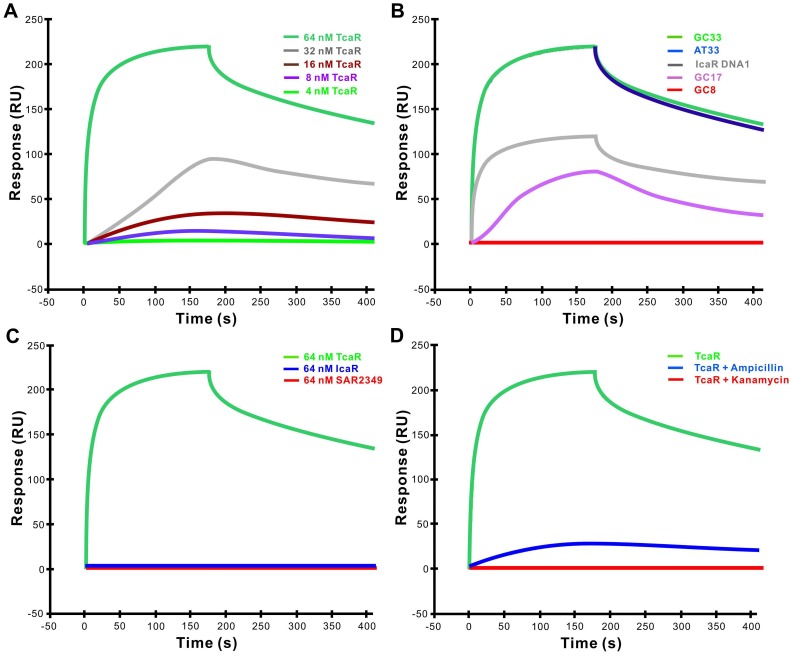
SPR sensorgrams of the binding of TcaR to DNA fragments at 25°C. (A) SPR sensorgram of the binding of varying concentrations of TcaR (4, 8, 16, 32, 64 nM) to biotin-labeled GC33 ssDNA (33mer GC-rich single-stranded DNA fragment) captured on SA chip with a ligand density of 120RU. The increase in RUs from the baseline was measured and used to calculated *k*_a_ and *k*_d_. One RU represents the binding of approximately 1 pg protein/mm^2^. (B) Comparison of SPR derived binding curves for different types of immobilized biotin-labeled DNA fragments interacting with TcaR protein (64 nM). The sensograms represent binding of, from bottom to top: GC8, GC17, IcaR DNA1, AT33 and GC33. The association (*k*_a_) and dissociation rate constants (*k*_d_) were derived by fitting the sensograms to a Langumir binding rate equations and are tabulated in Table 1. (C) The SPR sensorgram of the binding of different DNA binding proteins to the immobilized GC33 ssDNA fragment. The concentration of each target protein is 64 nM, followed by the same condition as used in other Biacore assays. (D) Sensorgram of the interaction between the immobilized GC33 ssDNA fragment and the TcaR protein (64 nM) in the presence of 640 nM kanamycin or ampicillin.

The binding affinity of GC33 and TcaR was determined quantitatively using surface plasmon resonance. Increasing concentrations of TcaR were passed across a flow cell coated with ssDNA GC33 and the binding response was recorded as changes in response units (RU) after subtraction of the binding response for the reference flow cell. Figure 2A shows a representative sensorgram. Analysis of the sensorgram data indicates *k*_a_ for the interaction of TcaR with the ssDNA GC33 is 8.8×10^5 ^M^−1^ s^−1^; *k*_d_ for the interaction of TcaR with the ssDNA GC33 is 9.5×10^−3^ s^−1^ (Table 1). To investigate whether TcaR binds to different types and different lengths of DNA molecules, Biacore experiment was used to test the binding of TcaR to DNA fragments of biotin-labeled ssDNA and hairpin DNA duplex (IcaR DNA1). Consistent with previous observations in Fig. 1B, TcaR binds to ssDNA GC33 and ssDNA AT33 with the higher affinity and the association rates of TcaR to IcaR DNA1, and ssDNA GC17, ranging from 4.3×10^5^ M^−1^ s^−1^ to 1.2×10^5^ M^−1^ s^−1^, are much lower (shown in Figure 2B). However, when look at a wider range of DNA sizes, TcaR showed no detectable binding ability to ssDNA 8-mer GC8 but the highest binding ability to ssDNA GC99. The association rate with ssDNA GC99 is 36-fold higher than with ssDNA GC33, along with a 20-fold higher off-rate, suggested that cooperative binding of TcaR may contribute significantly in its ssDNA binding activity (Table 1). Interestingly, the association rate of GC33 ssDNA is two-times higher than IcaR DNA1, but the dissociation rate of IcaR DNA1 is the lowest compared to the DNA fragments tested in this study, suggesting different modes of interaction occurs in ssDNA-TcaR and dsDNA-TcaR complex (Figure 2B).

**Table 1:** DNA-binding constants between TcaR dimer and the target DNA determined from SPR kinetic analyses.

Target DNA	*k*_a_ (M^−1^ s^−1^)	*k*_d_ (s^−1^)	*K*_a_ (M^−1^)^a^	Rmax
ssDNA (GC33)	8.8×10^5^	9.5×10^−3^	9.3×10^7^	507
ssDNA (GC17)	1.2×10^5^	4.3×10^−2^	2.8×10^6^	220
ssDNA (GC8)	-	-	-	
ssDNA (GC99)	1.6×10^6^	4.7× 10^−4^	3.4×10^9^	4354
ssDNA (AT33)	8.1×10^5^	9.3×10^−3^	8.7×10^7^	476
dsDNA (IcaR DNA1)	4.3×10^5^	5.0×10^−3^	8.6×10^7^	268
Control dsDNA	-	-	-	

a *K*_a_ value obtained from *k*_a_ divided by *k*_d_.

Furthermore, in order to determine whether other MarR family and TetR family proteins such as SAR2349 from *S. aureus* and IcaR from *S. epidermidis* have the ssDNA binding ability, we conducted a series of SPR experiments to analyze the binding ability of SAR2349 and IcaR proteins to GC33 ssDNA (Figure 2C). The result shows that not all MarR family proteins have this ssDNA binding ability, thus pointing to the specific ssDNA-binding feature of TcaR. In addition, we have previously demonstrated that antibiotics appear to antagonize the ssDNA binding activity of TcaR (Figure 1I). Therefore, a measurement for the effect of kanamycin and ampicillin to the GC33 ssDNA binding affinity of TcaR is conducted using surface plasmon resonance to confirm the result. As seen in Figure 2D, the affinity between TcaR and GC33 ssDNA is shown by a decrease in RU values in the presence of antibiotics. This was especially apparent with kanamycin, which yield the lower binding capacity. Taken together, we demonstrate that TcaR shows a higher binding affinity to ssDNA than to dsDNA, and several antibiotics could regulate the ssDNA binding activity of TcaR.

**Figure 3:**
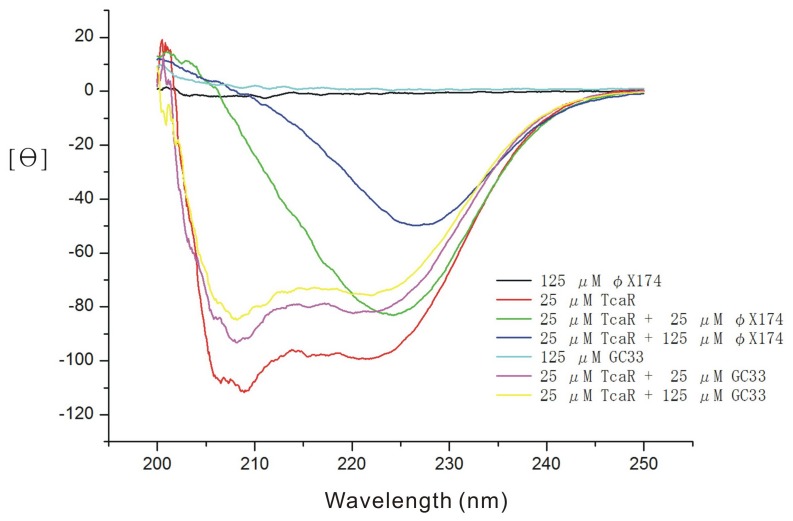
CD spectra of TcaR in various concentrations of GC33 and viral ssDNA φX174 (varying from 0 to 125 µM). TcaR concentration was 25 µM in a buffer of 20 mM Tris-HCl, pH 8.0, 150 mM KCl, 0.1 mM MgCl_2_, 0.05 mM EDTA, 12.5% Glycerol, 10 mM DTT. The protocols for CD data are described in the Experimental Procedures section.

Conformational changes of TcaR in response to ssDNA were monitored using CD spectroscopy [27]. As shown in Figure 3, the CD spectra of TcaR protein were scanned from 200 to 250 nm in the presence of GC33. With increasing concentrations of GC33 ssDNA, the CD spectrum shows a concomitant decrease in the intensity of the negative peak at 222 and 210 nm, revealing the conformational change of TcaR.

Furthermore, in order to examine whether TcaR shows a binding ability towards much longer ssDNA fragments such as viral φx174-ssDNA, the interaction between them was also examined with increasing concentration (0, 2.5, 10 mM) of viral ssDNA φX174. In the presence of viral ssDNA φX174, the spectral changes were particularly pronounced in the decreased intensity of the negative peak at 222 and 210 nm, with an ultimate diminished negative peak in the 225–230 nm region at high concentration of viral ssDNA φX174, which suggests strong interaction between viral ssDNA and TcaR. The results of the CD experiment reported herein provide strong evidence that TcaR exhibits strong binding affinity for viral ssDNA.

### TcaR Interacts with Circular Viral ssDNA

**Figure 4:**
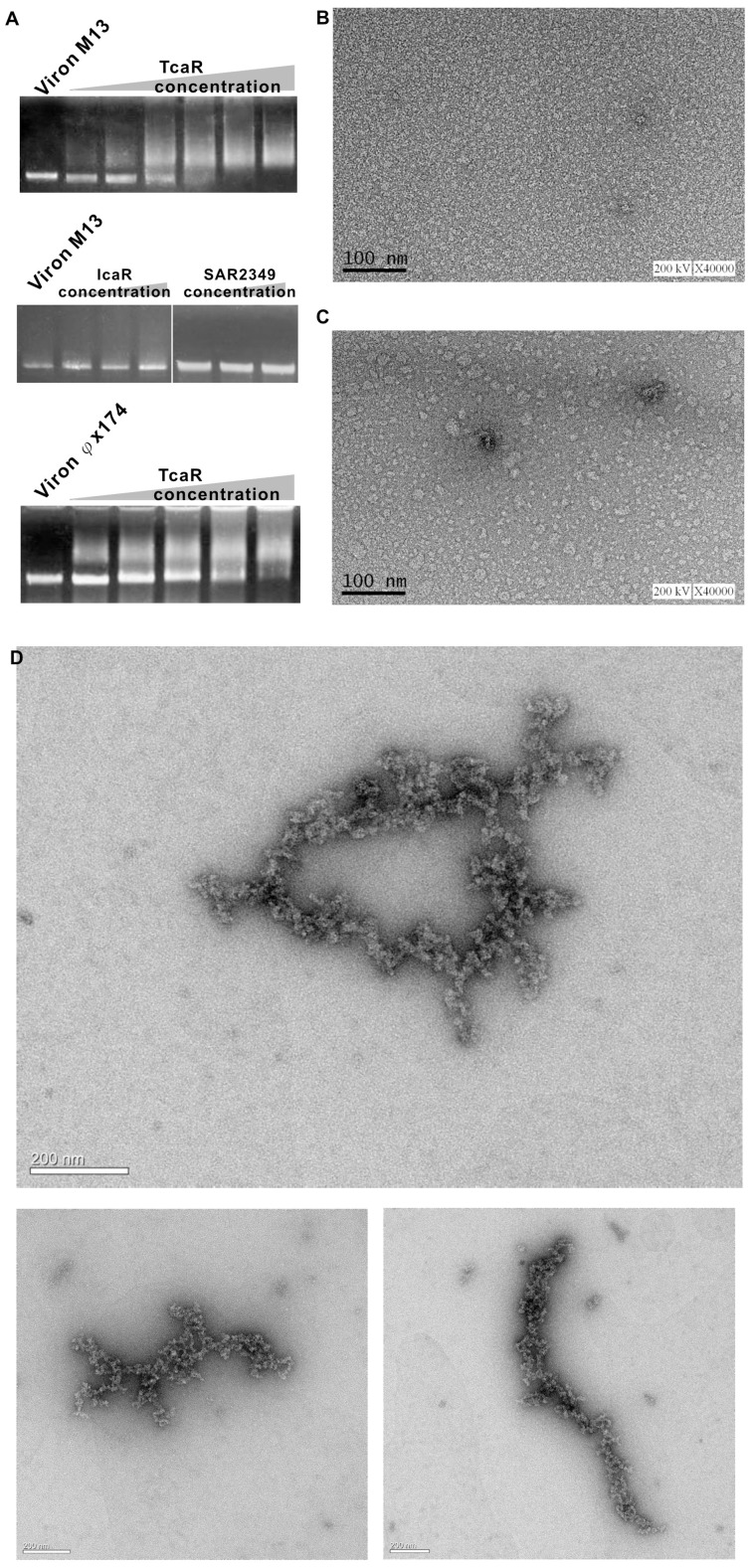
Analyses for TcaR-viral ssDNA complex. (A) EMSA analysis of the binding of TcaR and other DNA binding proteins to Viral M13 and φX174 ssDNA. Viral M13 and φX174 ssDNA (12 µM nucleotides) was incubated with an increasing concentration of TcaR, IcaR or SAR2349 at 30°C for 15 min. The reaction was analyzed by the same procedure as described in the legend to Fig 1B. (B–C) EM images of negative controls in TcaR protein only (0.3 µM) (B) and viral φX174 ssDNA only (12 µM) (C) (Scale bars = 100 nm). (D) EM image of TcaR-φX174 complex. The EM image of TcaR-φX174 (ssDNA) nucleoprotein filaments is shown. (Scale bars = 200 nm). See Materials and methods for details.

In order to further confirm our finding that TcaR forms complex with viral ssDNA, the M13 and φX174 phage ssDNA circles were used as probes in EMSA to evaluate TcaR binding. As shown in Figure 4A, TcaR reduced the mobility of the M13 and φX174 ssDNA, but *S. epidermidis* IcaR and *S. aureus* MarR family protein SAR2349 had no specific interactions with ssDNA. This indicated that TcaR has strong viral ssDNA-binding ability. It is also worth noting that other MarR family protein and TetR family protein do not have such ssDNA binding properties with high affinity, suggesting that the results from TcaR studies are not an artifact due to simple charge interactions between TcaR and ssDNA. Furthermore, since the attempt to obtain the TcaR-ssDNA complex structure was not successful, we resorted to EM analysis to image TcaR-φx174 complex and to pursue its 3-D reconstruction. After staining for 4 min with 2.5% uranyl acetate, EM analysis was performed with a Tecnai^tm^ G^2^ Spirit Bio TWIN (FEI CO., The Netherlands) using 120kV acceleration voltage. As seen in Figure 4B–D, EM imaging revealed that no complex was found in the negative control sections, whereas TcaR form a nucleoprotein filament with a circular viral φX174-ssDNA fully covered with proteins, suggesting strong and cooperatively interaction between viral ssDNA and TcaR. This is consistent with the EMSA results we observed. We are now testing another EM method as described by Lurz R et al. [28] to confirm the cooperative binding between the TcaR and viral φX174-ssDNA.

**Figure 5:**
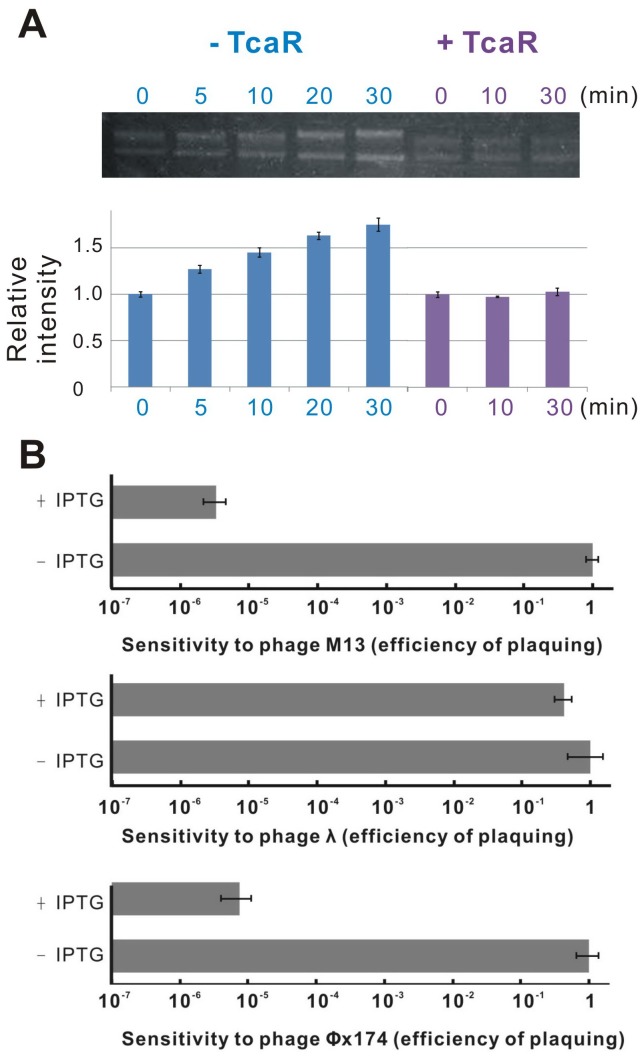
*In vitro* and *in vivo* assay for TcaR-viral ssDNA complex. (A) Replication assay of Viron M13-ssDNA in the absence or presence of TcaR protein. See Materials and methods for details. The fluctuations in machine sensitivity were corrected by normalizing all data to the control (reaction without the presence of TcaR at 0 min). Quantitative data is shown as mean±s.d, *n* = 3. (B) Effect of the presence of TcaR protein on the sensitivity of *E. coli* to different phages. Cells were transformed with engineered pET-16b-TcaR plasmid containing *lacI* gene and *lac* operator. The sensitivity of *E. coli* to each phage is represented as a histogram of the efficiency of plaquing, which is the plaque count ratio of a non-IPTG set to the IPTG set. Mean ± SD of plaquing data are shown (*n* = 3).

A distinct group of DNA-binding proteins called the ssDNA-binding proteins (SBP) could specifically bind ssDNA and be used in processes where the double helix is separated, including DNA replication, transcription, and recombination. Because TcaR is known as a MarR family transcription regulator that binds to specific dsDNA sequence with the winged helix-turn-helix (wHTH) DNA binding domain, the ssDNA binding ability of TcaR may not be involved in transcription. In order to clarify the role of TcaR-viral ssDNA interaction, our approach is to examine it with *in vitro* replication. We used single-primed M13 replication assay to measure the ability of purified TcaR protein to convert a primed single-strand M13 template to the duplex form in a manner that requires processive DNA synthesis. As seen in Figure 5A, M13-based in vitro DNA replication assay showed that the addition of TcaR protein to the reaction mixture, and incubation for up to 30 min, resulted in almost no DNA replication activity compared to controls. This indicated that TcaR markedly inhibits DNA replication and that the mechanism of inhibition, at least in part, involves interaction with viral ssDNA. These results suggest a possible role for TcaR in bacteriophage resistance.

Since 1980, investigators have developed an increasing number of bacteriophage therapies for the treatment, or prophylaxis, of bacterial infectious diseases [29], [30], [31]. Reports have described that appropriately administered phages can treat lethal infectious diseases caused by gram-negative and gram-positive bacteria, such as *Pseudomonas aeruginae*, *Klebsiella pneumoniae*, *Enterococcus faecium*, and *S. aureus* [32], [33], [34], [35]. Antibiotic resistance has become a global public health concern; thus investigators are extensively reevaluating phage therapies to fully exploit their antimicrobial potential [36], [37]. However, phages encounter a variety of different antiviral mechanisms during their infection of bacterial cells, such as prevention of phage adsorption and DNA entry, cutting of phage nucleic acids, and abortive infection systems [38]. Most reported antiphage systems have been shown to be relevant to the dsDNA phage, but not ssDNA, ssRNA, or dsRNA phages. To further confirm and clarify the first relationship between the TcaR protein and ssDNA phage resistance, the standard plaque assay was performed in *E. coli* as a model system since little is known about the ssDNA phage infecting *Staphylococci*. As seen in Figure 5B, induction of the TcaR protein in *E. coli* conferred increased host resistance to ssDNA phage (M13 and φX174) infection. However, a TcaR-expressing strain did not demonstrate reduced sensitivity to dsDNA phage Lambda (λ) infection, suggesting that the phage resistance was caused by TcaR-viral ssDNA complex. The observed biological differences point to a remarkable plasticity of TcaR. These findings, thus, may support a hypothesis that TcaR might interfere with viral ssDNA replication and establish a link between TcaR and ssDNA phage resistance.

### Conclusions

The MarR family transcriptional regulators serve as sensors of changing environments, allowing pathogenic bacteria to survive and persist in a dynamic environment [39]. However, up to now, the knowledge of MarR family protein-nucleic acid interaction has been focused on dsDNA and the MarR family protein-ssDNA interaction ability as well as their contribution to the multiple functions of TcaR is yet to be discovered. Better understanding of these interactions not only will benefit the understanding of many biological mechanisms but also is expected to provide a concept for designing a new therapy for *Streptococci*. In this work, we present the first attempt to investigate the TcaR-ssDNA interaction. The information of TcaR-ssDNA binding mode and the minimal binding length that we obtained from EMSA analysis and Biacore will be helpful for us to obtain TcaR-ssDNA complexed structure successfully. Moreover, we used *in vitro* replication assay and plaque assay to elucidate the specific biological role of the ssDNA binding ability of TcaR. Such observations may help us understand the mechanism of antibiotic resistance in the MarR family regulators.

## Materials and Methods

### Gene Cloning, Protein Production and Purification

The IcaR and TcaR proteins were expressed in *E. coli* BL21 (DE3) and purified as already described [17], [40]. The MarR homologous gene, SAR2349, was amplified directly from the *S. aureus* MRSA252 genome by polymerase chain reaction (PCR) and subsequently cloned into expression vector pET-32. This construct was transferred into *E. coli* of Arctic Express™ (DE3) RIL strain. The His_6_-tagged wild-type protein was over-expressed in Difco Luria-Bertani (LB) broth containing 50 mg/l ampicillin to an optical density at 600 nm of 0.5–0.6 and then induced with 0.5 mM IPTG (isopropyl-β-D-thiogalactopyranoside). Cells were grown for 2 days at 16°C. The cells were then harvested by centrifugation at 12,000 g for 30 min and disrupted by Constant Cell Disruption System (CONSTANT SYSTEM Ltd, UK) with lysis buffer containing 20 mM Tris-HCl (pH 8.0), 500 mM NaCl, and 20 mM imidazole. The homogenate was centrifuged at 27,000g for 30 min and the cell-free extract was loaded onto a Ni^2+^-NTA column, which had been previously equilibrated with lysis buffer. The column was washed with lysis buffer, and the His_6_-tagged SAR2349 was subsequently eluted by a linear gradient of imidazole from 10 mM to 500 mM. His-tagged SAR2349 eluted was dialyzed twice against 5 liters of buffer (20 mM Tris-HCl, pH 8.0, and 500 mM NaCl) and then subjected to Thrombin digestion to remove the tag. The mixture was then passed through another Ni^2+^-NTA column, and subsequently untagged SAR2349 protein was dialyzed twice against 3 liters of buffer (20 mM Tris-HCl, pH 8.0) and then passed through a Q-Sepharose anion-exchange column for further purification, and subsequently SAR2349 was eluted by a linear gradient of 10 mM to 500 mM NaCl-containing buffer and then dialyzed twice against 5 liters of buffer (20 mM Tris-HCl, pH 8.0, 150 mM NaCl, and 2 mM DTT) for storage. The purified SAR2349 protein was finally concentrated by 3 kDa cut-off size membrane of Amicon ultra-15 centrifugal filter units (Millipore, MA, USA) for storage at –80°C.

### EMSA Assay

The six oligonucleotide probes used in EMSA experiments were purchased from MDBio Inc. (Taiwan) (Figure 1A). The viron φX174 and M13 ssDNA were purchased from New England Biolabd (USA). For the preparation of double-stranded IcaR DNA1, equimolar amounts (100 µM each) of complementary oligonucleotides were mixed, fully denatured by heating at 95°C for 5 min in 10 mM Tris–HCl pH 8.0, 20 mM NaCl and allowed to cool gradually to room temperature. Gel shift assays were performed by incubating 1 µM of ssDNA or dsDNA with 1–4 µM purified recombinant proteins under binding conditions (20 mM Tris-HCl, pH 8.0, 150 mM KCl, 0.1 mM MgCl_2_, 0.05 mM EDTA, 12.5% Glycerol, 0.1 mM DTT and 1 mg/ml BSA) for 15 min at room temperature with gentle vortex. After incubation, 15 µl of the reaction solution was mixed with 3 µl of the sample loading dye and subsequently electrophoresed on 6% preequilibrated polyacrylamide gels in 1/2 Tris/acetate/EDTA (TAE) at 100 V for 30 min and visualized using SYBR Green I nucleic acid gel stain (Invitrogen). For competition assay, IcaR DNA1 probe duplex of 1 µM was pre-incubated with 2 µM TcaR (dimer) at room temperature for 15 min, then mixed with increasing concentration of GC33 ssDNA. In the assay for analyzing the effect of different antibiotics on the interaction between TcaR and ssDNA, 1 µM GC33 probe was pre-incubated with 2 µM TcaR (dimer) at room temperature for 15 min before mixing with 2 µM or 20 µM antibiotics.

### SPR-binding Assay

The affinity, association and dissociation between the drug and the DNA duplexes were measured using a BIAcore 3000A surface plasmon resonance (SPR) instrument (Pharmacia, Uppsala, Sweden) with a SensorChip SA5 from Pharmacia by monitoring the refractive index change of the sensor chip surface [41], [42], [43]. These changes are proportional to the amount of bound analyte. The SPR angle change is reported as resonance units (RU). Five fragments of 5′ biotinylated oligonucleotides probes purified by PAGE were purchased from MDBio Inc. (Taiwan). Activation buffer (100 mM NaCl, 50 mM NaOH) was injected for 1 min (20 µl) to remove any unbound streptavidin from the sensor chip. To control the amount of the DNA bound to the SA chip surface, 200 nM of the biotinated oligonucleotides were immobilized manually onto the surface of a streptavidin chip until 120 RU was reached in the first cell. The chip surface was then washed with 10 µl of 10 mM HCl to eliminate non-specific binding. The second flow cell was unmodified and served as a control. Different concentration of TcaR, IcaR, and SAR2349 proteins were injected at a flow rate of 30 µl/min in 50 mM Tris, 150 mM NaCl, pH 7.5 for 170 s to reach equilibrium. Blank buffer solution was then passed over the chip to initiate the dissociation reaction. At the end of each cycle, the surface was recovered with two 30 s injections of 0.025% SDS. SPR-binding constant is analyzed as described previously [44]. Sensorgrams for the interactions between DNA and TcaR were analyzed using BIA evaluation software to determine the association and dissociation rate constant (*k*_a_/*k*_d_). In the assay analyzing the effect of antibiotics on the interaction between TcaR and ssDNA, TcaR protein with 640 nM kanamycin or ampicillin in 50 mM Tris, 150 mM NaCl, pH 7.5 was injected on to the sensor chip.

### Circular Dichroism Spectroscopy

Circular dichroism (CD) spectra were obtained using a JASCO-815 CD spectropolarimeter. Temperature was controlled by circulating water at the desired temperature in the cell jacket. TcaR and DNA samples were prepared under the conditions identical to those prepared for EMSA assay. The CD spectra were collected between 250 and 200 nm with 1 nm bandwidth at 1 nm intervals. All spectra were obtained from an average of five scans. The photomultiplier absorbance did not exceed 600 V during the analysis. CD spectra were normalized by subtraction of the background scan with buffer alone. The mean residue ellipticity, [θ], was calculated based on the equation, [θ] = MRW×θλ/10×*l*×*c*, where MRW is the mean residue weight, θλ is the measured ellipticity in milidegrees at wavelength λ, *l* is the cuvette pathlength (0.1 cm), and *c* is the protein concentration in g/mL.

### Electron Microscopy (EM)

The TcaR proteins (0.3 µM) were first incubated at 30°C for 15 min in reaction buffer [20 mM Tris-HCl, pH 8.0, 150 mM KCl, 0.1 mM MgCl_2_, 0.05 mM EDTA, 12.5% glycerol, 10 mM DTT, 12 µM circular viron φX174 ssDNA (5386 nucleotides in length), 0.2 M ammonium acetate], and then chilled on ice to stop the reaction. The reaction product was diluted 100-fold with EM sample dilution buffer (2 mM MgCl_2_, 0.5 mM DTT, 10 mM HEPES pH 7.0). A droplet (4 µl) was placed for 1 min at room temperature on a copper grid (300 mesh, Pelco, USA) coated with fresh carbon. The excess buffer was then carefully blotted away from the edge of the grid with Whatman #1 filter paper (Whatman Inc., USA). After staining for 1 min with 2% uranyl acetate, excess liquid was removed and samples were dried at room temperature. Bio-transmission EM was performed with a Tecnai F20 Bio TWIN (FEI Co., Netherlands) using an acceleration voltage of 200 kV. Images were recorded with a slow scan CCD camera (Gatan MultiScan™ 600, USA) at a resolution of at least 4k×4k pixels.

### Replication Assay

For M13 replication assay, 250 µM of single primed M13mp18 ssDNA was incubated with/without 2 µM TcaR protein in the reaction mixture (20 µl) containing 25 unit Klenow fragment, 25 mM NaCl, 7 mM MgCl_2_, 1 mM EDTA, and 0.5 mM DTT for 3 min at 30°C to allow replication complexes to assemble at the primer template junction [45], [46]. Replication was allowed to proceed by addition of 60 µM dNTP. After incubation at 30°C for 30 min, the reactions were terminated by addition of 10 mM Tris-HCl, 5 mM EDTA, 0.5% SDS, and 50 µg of proteinase K (with total volume of 20 µl) and incubated at 50°C for 1 h. Conventional electrophoresis was then performed to verify the result of DNA replication (20-cm 0.8% agarose gel, 15 mA, 0.5X TBE buffer). The bands are visualized using SYBR Green I nucleic acid gel stain (Invitrogen) and quantified by Quantify One (BIO-RAD, USA).

### Phage Studies

Host sensitivity to phages was tested using a virulent variant of phage (M13, φX174 and γ) and *E. coli* BL21 (DE3) RIL transformed with engineered pET-16b-TcaR plasmid containing *lacI* gene and *lac* operator as host [47], [48], [49]. Cells were grown in LB media until the optical density (OD_600_) reached 0.6. TcaR protein was then induced by adding a final concentration of 0.1 mM IPTG and used in plaque assays as previously described [50], [51]. Plaque assays were performed in triplicate. Plates and top-agar contained LB and above mentioned concentrations of inducers. The sensitivity of the host to phage infection was calculated as the efficiency of plaquing, which is the plaque count ratio of a non-IPTG set to the IPTG set [52]. Error-bars were calculated as one standard deviation.

### Protein Data Bank Accession Codes

The atomic coordinates and structure factors for the TcaR-RNA complex have been deposited in the wwPDB with accession numbers of 4EJT.
